# Real-life study of dual therapy based on dolutegravir and ritonavir-boosted darunavir in HIV-1-infected treatment-experienced patients

**DOI:** 10.1371/journal.pone.0210476

**Published:** 2019-01-17

**Authors:** Elżbieta Jabłonowska, Ewa Siwak, Monika Bociąga-Jasik, Jacek Gąsiorowski, Anna Kalinowska, Ewa Firląg Burkacka, Kamila Wójcik-Cichy, Anna Piątek, Iwona Cielniak, Andrzej Horban

**Affiliations:** 1 Department of Infectious Diseases and Hepatology, Medical University of Lodz, Lodz, Poland; 2 Hospital for Infectious Diseases, HIV Out-Patient Clinic, Warsaw, Poland; 3 Department of Infectious Diseases, Jagiellonian University Medical College, Krakow, Poland; 4 Department of Infectious Diseases, Hepatology and Acquired Immune Deficiencies, Wroclaw Medical University, Wroclaw, Poland; 5 Hospital for Infectious Diseases, Medical University in Warsaw, Warsaw, Poland; University of Pittsburgh Centre for Vaccine Research, UNITED STATES

## Abstract

**Background:**

Dual therapy based on dolutegravir and ritonavir-boosted darunavir (DTG/DRV/r) is a combination of well-known drugs with a high genetic barrier to HIV resistance.

**Method:**

A retrospective analysis of all HIV-1 infected treatment-experienced patients who switched to DTG/DRV/r from May 2014 till March 2017 in 4 Polish centres–results of a 48-week treatment.

**Results:**

The study group consisted of 59 men and 17 women. Median baseline parameters were: age– 42.7 years, CD4 cells count– 560.5 cells/μl, CD4 cells nadir– 150 cells/μl, *number* of prior antiretroviral *regimens–* 3. The introduction of dual therapy was primarily due to virologic failure (30 patients), adverse events on previous regimens (17 patients) and therapy simplification (27 patients). At week 48 the treatment *was continued* in 70/76 of patients and the median CD4 cells count increased from 560.5 to 641.0 cells/μl. The therapy was discontinued in six patients (1 –virologic failure, 1 –decrease of estimated glomerular filtration rate (eGFR), 1 –myalgia, 3 –lost to follow-up). At week 48 six patients had detectable viremia, but only in one patient viremia was higher than 200 copies/ml. At week 48 the level of serum total cholesterol of the investigated subjects was statistically significantly higher than at the moment of dual therapy introduction (185.8 mg/dl vs. 174.8 mg/dl- p<0.05). However, in patients previously not treated with TDF, there were no changes in lipid parameters during therapy. *Proteinuria* was observed in 13.2% of patients before the switch to dual therapy and in 7.1% of patients at week 48.

**Conclusions:**

The investigated dual therapy was effective and safe. The observed increase in lipid parameters only concerned the patients who had used a TDF-based regimen prior to analysed dual treatment.

## Introduction

Due to the introduction of combined antiretroviral therapy (cART), persons infected with the Human Immunodeficiency Virus (HIV) are currently able to live almost as long as non-infected subjects. The therapy increases the number of CD4+T lymphocytes (CD4 cells), which is the key factor determining life expectancy of HIV-positive people [1]. Considering that the introduction of cART is recommended for all HIV-infected patients [2, 3], also young people with a high CD4 cells count are subject to a lifelong treatment. In the era of modern cART, which includes very potent drugs with improving tolerability profiles, the choice of an optimal combination becomes more and more dependent on adverse events, which may occur in the long term.

For more than twenty years, triple drug combinations have remained a standard of care for HIV infection. Current guidelines recommend a combination of two backbone drugs–namely abacavire (ABC) and lamivudine (3TC) or tenofovire (TDF or TAF) and emtricitabine (dTC)–with a third drug from another antiretroviral (ARV) group, for all patients who start treatment [2, 3].

At the same time, long experience with triple combinations shows not only their benefits in terms of antiviral efficacy, but also the associated side effects [2, 4–10]. Indeed, both backbone regimens are known of long-term cumulative adverse events. Tenofovir can cause proximal renal tubulopathy, reduce the glomerular filtration rate (GFR) and decrease the bone mineral density [4, 5, 11, 12], while abacavir can increase the risk of cardiovascular diseases [13–15]. Moreover, since both drugs belong to a group of nucleoside/nucleotide reverse transcriptase inhibitors (NRTIs), and are thus characterized by a low genetic barrier to resistance [16], the recommended regimens may not be suitable for patients in whom such resistance occurs. Both factors highlight the importance of sharing the experience with non-standard regimens across the medical community.

## Aim

The study aimed to evaluate the efficacy and tolerability of the dual therapy containing dolutegravir and ritonavir-boosted darunavir (DTG/DRV/r).

## Material and methods

Retrospective analyses of medical records were performed in four Polish HIV centres with a view to identifying the number of HIV-1 infected treatment-experienced patients who switched to DTG/DRV/r from May 2014 till March 2017. All patients who fulfilled these conditions were included into the study. Medical data from the moment of dual therapy introduction and at the 48 week of treatment were collected and assessed.

To evaluate the use of the DTG/DRV/r regimen in a real-world setting, the exclusion criteria were not established. Notwithstanding the above, no mutations associated with DRV or DTG resistance had been confirmed in the investigated patients before the dual therapy was introduced. However, in most studied patients, the integrase genotypic testing was not performed. The virologic failure of this therapy before 48 week of treatment was defined as stopping dual therapy because of lack of virological response—doctor decision or viral load above 50 copies/ml at 48 week. Effectiveness of dual therapy was measured as a proportion of patients continuing this treatment throughout the investigated period of 48 weeks.

The measurements of lipid parameters, CD4 cells count, estimated glomerular filtration rate (eGFR) and proteinuria performed at two time points–before the introduction of DTG/DRV/r therapy (maximum 3 months) and after 48 weeks of dual treatment (±8 weeks)–formed the basis of the relevant comparisons. The assessment of GFR was performed using the Modification of Diet in Renal Disease Study formula [17]. The CD4 cells count was established by means of flow cytometry. Creatinine concentration and HIV viremia were determined using the kinetic compensated Jaffe method and RT-PCR method (Cobas AmpliPrep/Cobas TaqMan HIV-1 Test, Roche Diagnostics), respectively. To assess proteinuria dipstick analyses were performed. The levels of serum total cholesterol (TC) and triglycerides (TG) were measured using traditional enzymatic methods. The Low-density lipoprotein cholesterol (LDL-C) fraction was estimated by means of Friedewald formula, provided that the level of triglycerides did not exceed 400 mg/dl. If this was not the case, the measurements of LDL-C were performed by direct methods.

The study was approved by the ethics committee (Medical University of Lodz). All patients signed the informed consent form. All data were analysed anonymously.

## Statistical methods

The differences in CD4 cells count, TC, LDL-C and TG before the introduction of the dual therapy and at 48 weeks of treatment were assessed by means of paired sample T test or Wilcoxon signed-rank test. For categorical parameters, such as the presence of proteinuria and eGFR<60ml/min/1.72m^2^, the McNemar test was used.

## Results

### Study group

The study group consisted of 76 HIV-1-infected patients (59 males and 17 females) with a median age of 42.7 years. Homosexual and bisexual contacts were identified as the main route of HIV transmission. All subjects were treatment-experienced with the median number of prior antiretroviral regimens equal to 3. At the moment of dual therapy introduction, the median CD4 cell count was 560.5 cells/μl. The parameters of the study group are presented in Table 1. The regimens administered directly before the switch to DLT/DRV/r treatment are presented in Table 2.

**Table 1:** Characteristics of study group.

		n	%
Men		59/76	77.6
Routes of HIV transmission	Intravenous drug use	19/76	25.0
	Heterosexual	11/76	14.5
	Homosexual/bisexual	44/76	57.9
	Other/unknown	2/76	2.6
		MEDIAN	LQ-UQ
Age at the moment of HIV infection diagnosis (years)		33.6	27.5–40.7
Age at the moment of the introduction of dual therapy		42.7	37.8–52.0
Lymphocyte CD4+ at the moment of the introduction of dual therapy (cells/μl)		560.5	362.5–756.3
Lymphocyte CD4+ nadir (cells/μl)		150.0	80.5–328.0
N*umber* of prior antiretroviral *regimes*		3	2–5

**Table 2:** ARV regimens directly before the switch to DLT/DRV/r.

Type of therapy	N (%)
Standard three-drug combination with two NRTIs and one boosted PI	39
Standard three-drug combination with two NRTIs and one NNRTI	4
Standard three-drug combination with two NRTIs and one InI	4
Two- drug combination	19
Non-standard three-drug combination	6
Four-drug combination	4

NRTI—nucleoside/nucleotide reverse transcriptase inhibitors

NNRTI-non-nucleoside reverse transcriptase inhibitorsPI- protease inhibitors

InI- integrase inhibitors

### The reasons for the introduction and for the discontinuation of DLT/DRV/r treatment

The DTG/DRV/r therapy was most commonly introduced as a response to a virologic failure on previous regimens. In 27 patients the reason for the switch to DTG/DRV/r was simplification of the treatment. A combination, which had been replaced most frequently for this reason, was a regimen based on RAL/DRV/r with a twice-daily RAL dosing (14 patients).

All reasons for the introduction of DTG/DRV/r are presented in Table 3.

**Table 3:** Reasons for the introduction of DTV/DRV/r.

Reason		n
Adverse events n = 17	Osteopenia/osteoporosis	3
	Kidney disorder	5
	Hematologic disorders	1
	*Hypersensitivity reaction* to NRTIs	1
	Acidosis	1
	Polineuropathy	2
	Pancreatitis	1
	Other intolerance	3
Treatment failure n = 30	Virologic failure	30
Other reasons n = 29	Simplification of therapy	27
	Other	2
Total		76

**Fig 1:**
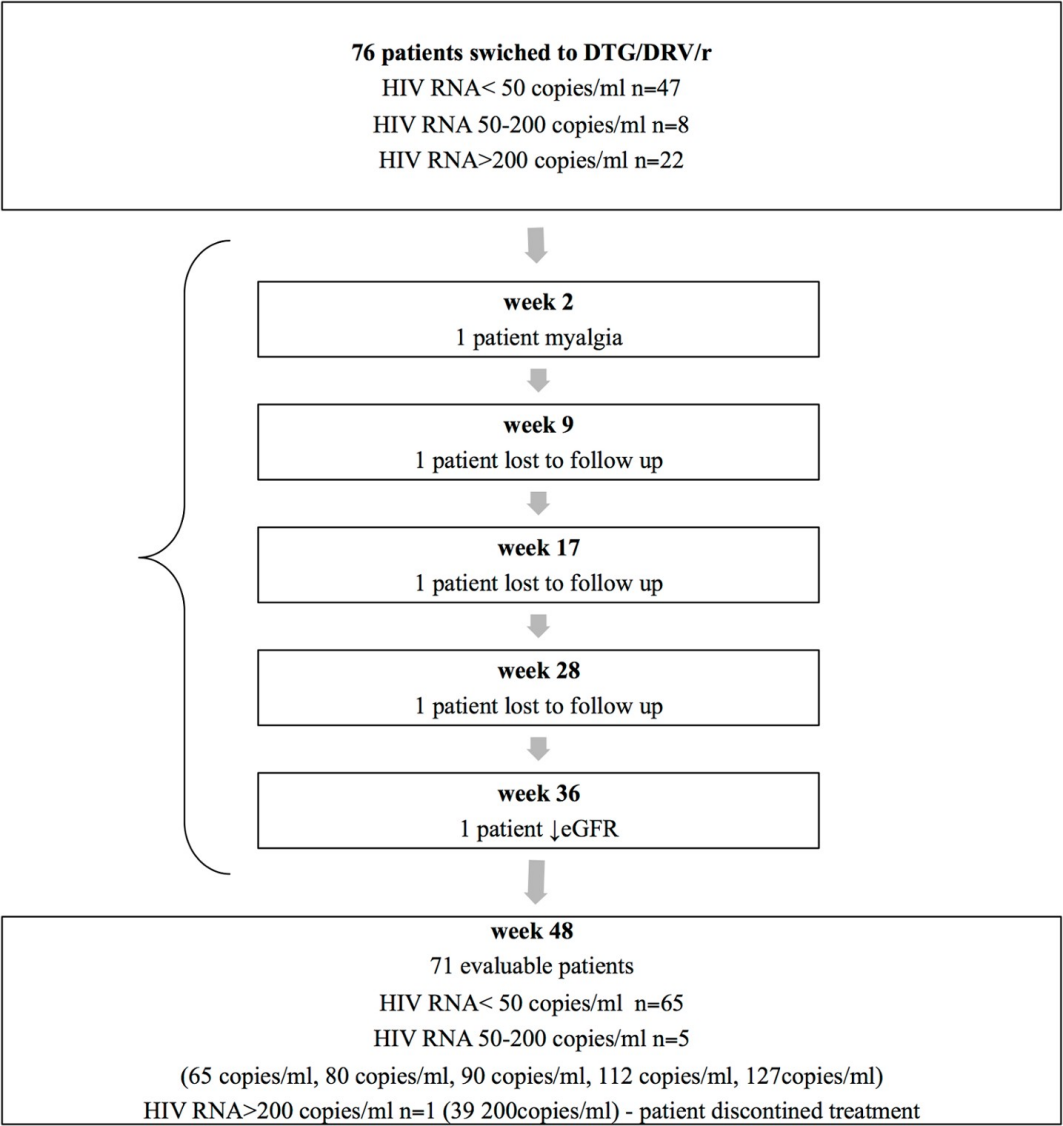
Algorithm of the study population. DTG/DRV/r = dolutegravir and ritonavir-boosted darunavir.

The dual therapy was stopped in six patients, one of whom experienced virologic failure (at week 48). Three patients were lost to follow-up before 48 weeks of treatment–in these patients viremia was not assessed during the dual therapy. In two patients adverse events were the reason for stopping dual therapy (Fig 1).

### Virologic response

At 48 weeks (+/- 4 weeks) viral load was above 50 copies/ml in six patients, of whom only in one subject the threshold of 200 copies/ml was exceeded. The treatment was discontinued in this patient, even though no mutations in the genotyping test were found. Five patients with viremia lower than 200 copies/ml remained on DTG/DRV/r therapy.

### The assessment of immunologic failure, renal safety and lipid profile after 48 weeks of dual therapy (+/- 6 weeks)

**Fig 2:**
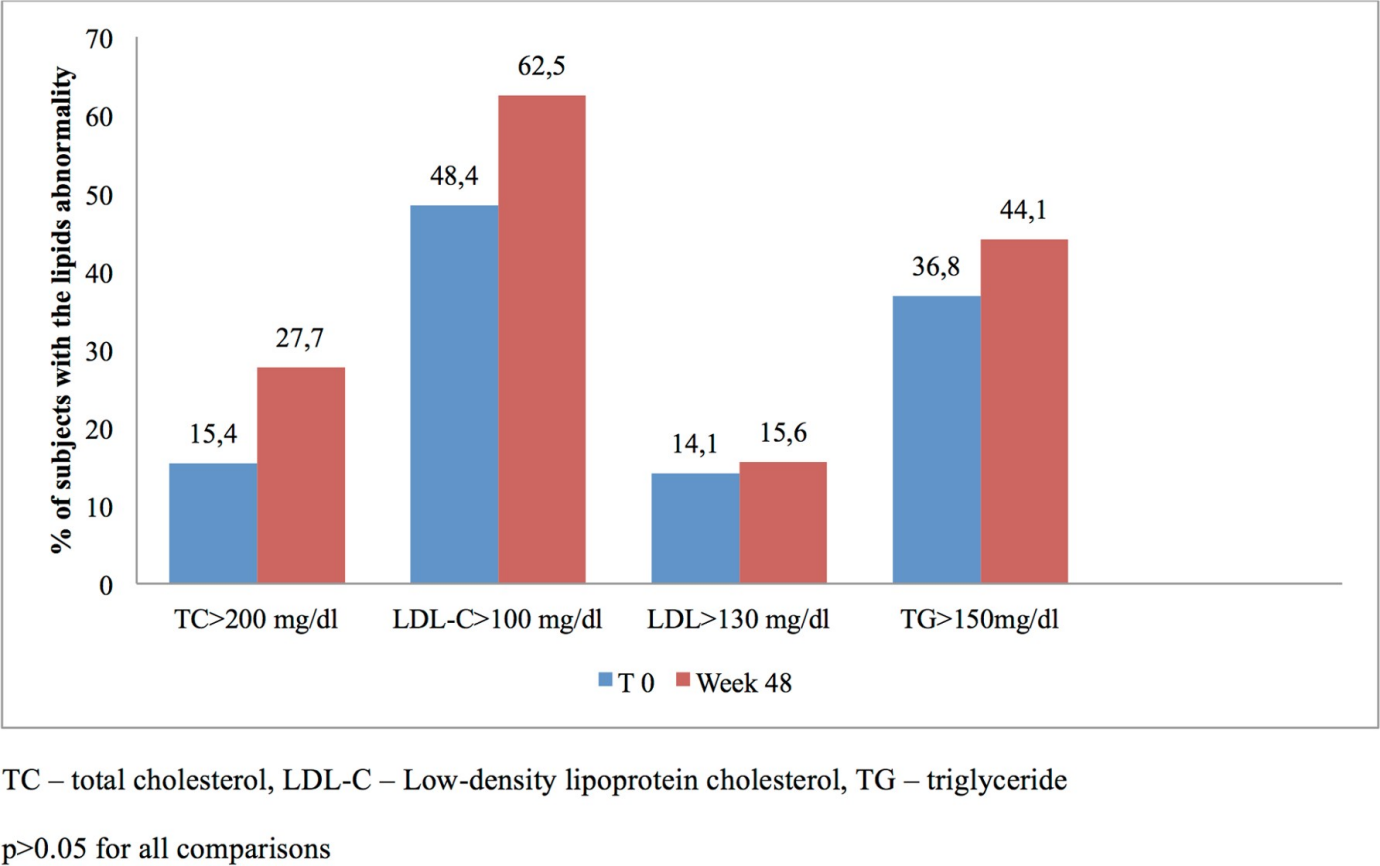
Number of subjects with lipids abnormality at baseline and week 48 TC -total cholesterol, LDL-C—Low-density lipoprotein cholesterol, TG -triglyceridep>0.05 for all comparison.

We found a statistically significant increase in the CD4 cells count (median 80.5 cells/μl) and in TC level. By contrast, no statistically significant changes were found in LDL-C and TG levels (Table 4). The number of subjects with lipids abnormality at baseline and at week 48 is presented in Fig 2. Proteinuria was observed in 13.2% of patients before the switch to dual therapy and in 7.1% at week 48 (p>0.05); eGFR<60 mL/min/1.72m^2^ was noted in 10.5% before the switch to dual therapy and in 12.7% at week 48 (p>0.05).

**Table 4:** Comparison of results before the introduction of DTG/DRV/r therapy and at 48 weeks of treatment.

	Before the introduction of DLT/DRV/r		48 weeks of treatment		P
	MEDIAN (LQ- UQ)	Mean ±SD	MEDIAN (LQ- UQ)	Mean ±SD	
Lymphocyte CD4+ (cells/μl)	560.5 (362.5–756.3)	560.7±276.6	641.0 (433.5–801.5)	639.6±280.6	<0.0001
Lymphocyte CD4+/CD8+	0.7 (0.4–0.9)	0.7±0.3	0.7 (0.5–0.9	0.7±0.3	<0.0001
Total cholesterol (mg/dL)	174.8 (150.2–192.3)	173,8±34.9	185.8 (161.3–200.8)	186.5±36.1	<0.05
Cholesterol LDL (mg/dL)	98.7 (77.6–119.9)	97.37±29.4	103.7 (83.7–119.1)	101.6±27.8	>0.05
Triglyceride (mg/dL)	132.0 (90.0–185.1)	159.3±97.6	138.1 (100.0–202.7)	170.6±108.8	>0.05

Analysis of the factors which could have affected the increase of cholesterol levels Medical records of the study group in the analysed period did not find any of the patients who had taken cholesterol-lowering drugs to have interrupted such treatment or begun alcohol abuse. At week 48, statin intake was commenced in 1 patient, 5 patients were diagnosed with alcohol abuse, yet this had already been the case before the switch of the treatment, and 2 patients were reported to quit smoking.

Since a positive impact of TDF on lipid parameters was found in other studies, in order to assess whether the observed increase in cholesterol levels was related to the discontinuation of this drug, a targeted analysis of lipid concentrations in the study group was performed. For purpose of this assessment a distinction was made between patients who had taken TDF as part of their previous regimens (45 subjects) and patients who had not taken this drug prior to the introduction of the analysed dual treatment (31 subjects). The increase in total cholesterol level and LDL cholesterol at 48 weeks of treatment was found in patients with previous TDF intake. No changes in lipid parameters were observed in the second subgroup (Table 5).

**Table 5:** Lipids changes during therapy in subgroups according to the prior TDF intake.

Patients treated with TDF					
	Before the introduction of DTG/DRV/r		48 weeks of treatment		P
	MEDIAN (LQ- UQ)	Mean ±SD	MEDIAN (LQ- UQ)	Mean ±SD	
Total cholesterol (mg/dL)	179.0 (142.8–190.0)	171.7±35.3	185.7 (158.7–199.2)	181.8±34.3	<0.01
Cholesterol LDL (mg/dL)	101.5 (67.0–119.6)	93.5±30.9	103.7 (88.0–115.2)	101,8±24,8	<0.05
Triglyceride (mg/dL)	113.1 (88.0–172.8)	143.6±93.1	115.6 (90.0–177.0)	156.5±113.7	>0.05
Patients treated without TDF					
Total cholesterol (mg/dL)	173,4 (162.1–197.4)	176.4±35.0	188.08 (164.9–202.0)	192.4±38.1	>0.05
Cholesterol LDL (mg/dL)	99.85 (85.9–122.3)	102.1±27.1	106.81 (77.8–126.9)	101.5±31.5	>0.05
Triglyceride (mg/dL)	136.40 (113.0–227.4)	178.4±101.1	155.0 (109.8–233.8)	187.8±101.8	>0.05

## Discussion

Despite the fact that NRTIs can induce the mitochondrial toxicity three-drug combinations based on NRTIs have remained a preferred regimen for the treatment of HIV-infected patients since at least two decades [2, 3]. However nowadays a growing number of studies focuses on dual regimens, which do not include NRTIs [18–24]. In our previous study the efficacy and safety of a dual regimen based on raltegravir (RAL) and DRV/r was analysed. The regimen was found to be effective in most patients, yet the inconvenience of having to administer drugs twice per day resulted in a switch of therapy to DTG/DRV/r in some of the studied subjects. When we realized that there were more patients taking this combination of drugs in different Polish centres, we decided to retrospectively analyse the results of such treatment.

When a new combination of drugs is introduced there are many concerns about its safety and efficacy. Theoretically a combination composed of DTG/DRV/r should be more effective than other dual therapies because of a high genetic barrier to resistance of both drugs [9, 25, 26]. However, not only the strength of individual components, but also other factors can have an influence on the safety and efficacy of a regimen. Fortunately pharmacokinetic data show adequate steady-state drug concentrations of these drugs combined together [27]. In reference to safety and tolerability of the investigated combination the same risks as those connected with its individual components can be expected, in addition to which other unexpected issues may occur. DTG is known of an excellent tolerability profile but lately raised some concerns about neurological side effects [8, 9, 28]. DRV/r, like other protease inhibitors, is known of an unfavourable metabolic profile, but is still the most favourable one within this group [9].

Our results indicate that a combination of DTG and DRV/r is well tolerated and effective, as demonstrated by the fact that 92.1% of patients remained on therapy throughout the investigated period of 48 weeks. It is worth stressing that in one patient, in whom the therapy was discontinued because of virologic failure, no mutations associated with DRV or DTG resistance were found. A high virologic response and a good tolerability was also found in an Italian study where 90.8% of investigated patients who switched to DTG/DRV/r had viral load below 50 copies/ml at week 48 of the treatment [19].

Further attention is drawn to the heterogeneity of our study group along with the previous ARV experience of all investigated subjects. Indeed, the study was not limited to patients in whom the dual regimen was introduced due to a virologic failure or adverse events on previous regimens. It also encompassed individuals who had previously been subject either to a salvage therapy, or to a dual therapy based on RAL/DRV/r as mentioned above. While the aforementioned situations do not exhaust all possible reasons for the introduction of DTG/DRV/r, they certainly reveal the heterogeneity of the studied population as reflected in our study group. In our view this heterogeneity is not a limitation, but rather an advantage of this non-interventional study as it shows the efficacy and safety of the analysed dual combination in a real life setting.

In our study, after the switch to the dual regimen a lower incidence of proteinuria was found, while an increase of eGFR during therapy was not established. It is known that DTG may cause an increase in creatinine concentrations as a result of inhibiting organic cation transporter 2 and a consequent reduction in tubular secretion of creatinine. This increase is, however, not associated with a decrease of GFR [9].

We did not find central nervous system adverse events to be the reason for therapy discontinuation, which can imply that such events either did not occur, or occurred with low intensity. The only negative impact of the new regimen was a statistically significant increase in the total cholesterol level. Nevertheless, this did not lead to a change of the administered regimen and was observed only in patients who had previously taken TDF. The increase in lipid parameters was also observed in other studies in which the switch from TDF to a different drug was investigated. The mechanism in which TDF reduces cholesterol concentration is not clear [29].

Our observational study has limitations, including the lack of a control group; however, our results suggest that DTG with DRV/r can present a viable option for selected groups of ARV-experienced patients.

In summary, the investigated dual therapy was effective and safe. In patients previously treated with TDF switching to DTG/DRV/r can cause an increase in total and LDL cholesterol levels.

## Supporting information

S1 Dataset Virological response and history of antiretroviral treatment. (XLSX)
